# The 5′UTR-intron of the *Gladiolus* polyubiquitin promoter *GUBQ1* enhances translation efficiency in *Gladiolus* and *Arabidopsis*

**DOI:** 10.1186/1471-2229-12-79

**Published:** 2012-06-06

**Authors:** Kathryn Kamo, Ah-Young Kim, Se Hee Park, Young Hee Joung

**Affiliations:** 1 U.S. Department of Agriculture, Floral and Nursery Plants Research Unit, Beltsville, MD, 20705-2350, USA; 2 School of Biological Sciences and Technology, Chonnam National University, Gwangju, 500-757, South Korea

## Abstract

**Background:**

There are many non-cereal monocots of agronomic, horticultural, and biofuel importance. Successful transformation of these species requires an understanding of factors controlling expression of their genes. Introns have been known to affect both the level and tissue-specific expression of genes in dicots and cereal monocots, but there have been no studies on an intron isolated from a non-cereal monocot. This study characterizes the levels of GUS expression and levels of *uidA* mRNA that code for β-glucuronidase (GUS) expression in leaves of *Gladiolus* and *Arabidopsis* using *GUBQ1*, a polyubiquitin promoter with a 1.234 kb intron, isolated from the non-cereal monocot *Gladiolus*, and an intronless version of this promoter.

**Results:**

*Gladiolus* and *Arabidopsis* were verified by Southern hybridization to be transformed with the *uidA* gene that was under control of either the *GUBQ1* promoter (1.9 kb), a 5′ *GUBQ1* promoter missing its 1.234 kb intron (0.68 kb), or the CaMV 35 S promoter. Histochemical staining showed that GUS was expressed throughout leaves and roots of *Gladiolus* and *Arabidopsis* with the 1.9 kb *GUBQ1* promoter. GUS expression was significantly decreased in *Gladiolus* and abolished in *Arabidopsis* when the 5′UTR-intron was absent. In *Arabidopsis* and *Gladiolus*, the presence of *uidA* mRNA was independent of the presence of the 5′UTR-intron. The 5′-UTR intron enhanced translation efficiency for both *Gladiolus* and *Arabidopsis*.

**Conclusions:**

The *GUBQ1* promoter directs high levels of GUS expression in young leaves of both *Gladiolus* and *Arabidopsis*. The 5′UTR-intron from *GUBQ1* resulted in a similar pattern of β-glucuronidase translation efficiency for both species even though the intron resulted in different patterns of *uidA* mRNA accumulation for each species.

## Background

There are many non-cereal monocots of agronomic (garlic, onions, banana, sugarcane, millet), horticultural (turf and forage grasses such as tall fescue, Kentucky bluegrass, creeping bentgrass, perennial ryegrass, St. Augustinegrass, buffalograss, *Zoysiagrass japonica*, *Paspalum vaginatum*, *Paspalum notatum*, floral crops such as orchids, lilies, tulips, crocus, amaryllis, iris), and potential biofuel (switchgrass, *Miscanthus*) importance. Successful transformation of these species requires an understanding of how their gene expression is controlled by promoter elements such as introns.

Introns have been found to significantly stimulate gene expression. Dicot introns have been used to transform dicot plants, and levels of intron-mediated expression (IME) in leaves ranged from 2–20× [1-4]. Monocot introns have been shown to enhance gene expression in monocots 20–100× [5-10]. Most of the studies with monocots have been transient transformation studies using callus cells or protoplasts of cereal monocots rather than stable transformation of plants to characterize expression within specific plant tissues. There have been a few transient transformation studies showing IME of non-cereal monocots (banana, lily, bluegrass) using introns (maize *Ubi1Arabidopsis UBQ*) isolated from either cereal monocots or dicots [11-14]. Half of the introns tested by Vain et al. [12] showed different results on transient expression for maize and bluegrass indicating a difference between cereal and non-cereal monocots.

This is the first study using an intron isolated from the promoter of a non-cereal monocot and evaluating its effect on expression in both a dicot and a monocot plant. Previously we had reported that the non-cereal intron from the *GUBQ1* promoter from *Gladiolus* enhanced GUS expression in suspension cells [15]. It is critical to verify IME using transformed plants as IME has been shown to differ dramatically depending on the tissue it is expressed in [4,16,17]. The sugarcane ubiquitin promoter including its intron expressed GUS at high levels in sugarcane callus, but there was no expression in plants of sugarcane [18]. Results in this study show that IME occurs in both a dicot and non-cereal monocot plant with the *GUBQ1* intron. This information will contribute to our understanding of factors controlling gene expression in a non-cereal monocot and can be used when genetic engineering applications require high levels of expression.

## Results

### Southern hybridization

**Figure 1:**
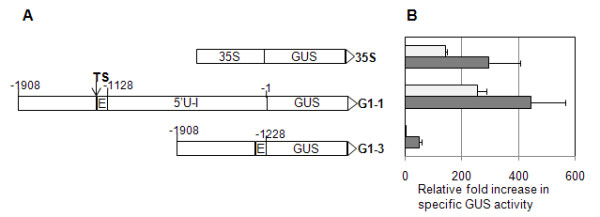
**A. Diagram of the DNA constructs used for transformation.*Gladiolus GUBQ1*promoter (G1-1) and an intronless version (G1-3).** TS, transcription start; E, 5′UTR-exon; 5′U-I, 5′UTR intron; 35 S, CaMV 35 S promoter. Negative numbers represent the nucleotides on the 5′ side of the translation start codon. For *Gladiolus* transformation pUC-GUS was used as the backbone vector, pCAMBIA1391Z for *Arabidopsis* transformation, and pBI121-GFP for transient transformation of tobacco. **B**. Relative fold increase in the specific GUS activity for leaves from *Arabidopsis* (white bars) and *Gladiolus* plants (shaded bars) transformed with either the CaMV 35 S, G1-1, or G1-3 constructs. Three independently transformed lines were used for each of the constructs analyzed for GUS expression. Standard error shown.

**Figure 2:**
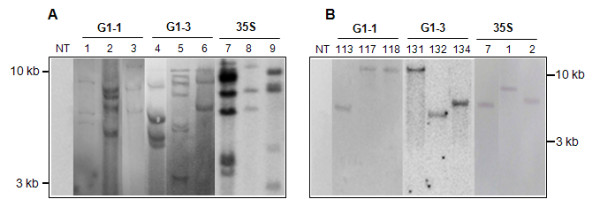
**DNA blots of genomic DNA isolated from (A)*Gladiolus*and (B)*Arabidopsis*plants containing the*uidA*gene under control of either the CaMV 35 S (35 S), G1-1, or G1-3 promoter.** Genomic is digested with *Hind* III and hybridized with a 511 bp *uidA* specific probe. Numbers indicate the transgenic plant lines or non-transformed (NT) plant. Two size markers, 3 and 10 kb, are shown.

*Gladiolus* and *Arabidopsis* plants were transformed with the *uidA* gene under control of either a 1.9 kb *GUBQ1* promoter (G1-1), a 5′end of *GUBQ1* that is lacking the intron (G1-3), or the CaMV 35 S promoter (Figure 1). Southern hybridization confirmed integration of the *uidA* gene in *Gladiolus* plants (Figure 2A). *Hind* III used for digesting the genomic DNA of *Gladiolus* and *Arabidopsis* cuts once immediately 5′ of the *GUBQ1* or CaMV 35 S promoter region. Three plants of *Gladiolus* that were shown by the Southern to be independently transformed had 2–5 copies of the *uidA* gene under control of G1-1. *Gladiolus* plant lines transformed with the *uidA* gene under control of either G1-3 or CaMV 35 S were also multicopy with 4–7 and 3–7 copies, respectively, of the transgene.

Approximately 30% of the transformed *Arabidopsis* plants contained a single copy of the *uidA* gene, and only those with a single copy of the *uidA* gene were used in this study (Figure 2B).

### GUS expression

**Figure 3:**
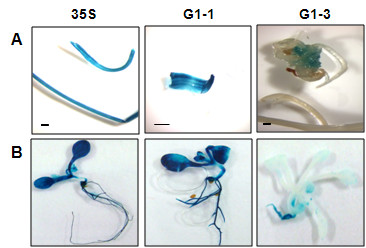
**Histochemical GUS staining of transgenic (A)*Gladiolus*and (B)*Arabidopsis*plants containing the*uidA*gene under control of either the CaMV 35 S (35 S), G1-1 or G1-3 promoter.** The *Gladiolus* plant material was taken three weeks after subculture from plantlets growing in vitro on MS medium. Plant tissue was incubated 16 h at 37°C in the staining solution. Magnification bars in A represent 2 mm.

**Figure 4:**
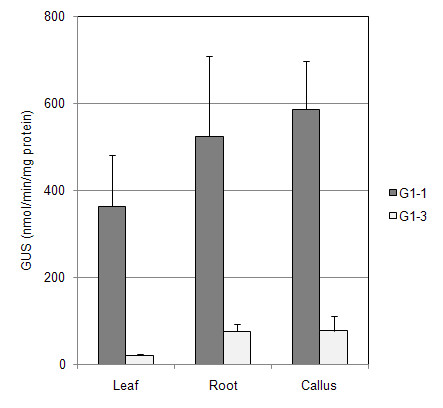
**Expression of the*uidA*gene in transgenic*Gladiolus*organs and callus.** GUS specific activity was measured using three plants for each of the three transformed lines, and the mean and standard error are shown.

Histochemical staining showed GUS expression throughout the leaves of transgenic *Gladiolus* plants with the CaMV 35 S and G1-1 promoters (Figure 3A). High levels of specific GUS activity were comparable with the *GUBQ1* and CaMV 35 S promoters in leaves of *Gladiolus* from plants transformed with either the G1-1 or 35 S promoters (Figure 1B). Specific GUS activity measured in leaves of transgenic *Gladiolus* plants with G1-3 was significantly lower than that of G1-1 plants indicating the importance of the intron on expression (Figure 1B). GUS expression was not evident from histochemical staining in leaves and roots of *Gladiolus* plants transformed with the G1-3 promoter, and light blue staining indicative of GUS expression was present in callus found at the basal meristem region of G1-3 plants (Figure 3A). A quantitative comparison of GUS activity in leaf, root, and callus confirmed the low level of GUS expression in both types of organs and callus tissue with the G1-3 promoter and the high level of expression with the G1-1 promoter (Figure 4).

*Arabidopsis* showed higher levels of specific GUS activity with the *GUBQ1* promoter than the CaMV 35 S promoter (Figure 1B). Histochemical staining showed GUS expression throughout the entire leaves and roots of transgenic *Arabidopsis* plants with the CaMV 35 S and G1-1 promoters (Figure 3B). Staining occurred at the periphery in some of the leaves of *Arabidopsis* that had the G1-1 and G1-3 promoters. The blue staining was considerably lighter in *Arabidopsis* transformed with the G1-3 promoter as compared to the dark blue staining seen in G1-1 plants. The specific GUS activity was negligible in leaves with the G1-3 promoter confirming the importance of the *GUBQ1* intron to expression in *Arabidopsis* (Figure 1B).

### RNA and translation efficiency

**Figure 5:**
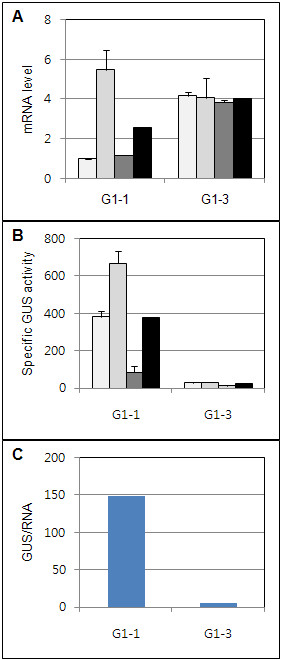
**Expression of the*uidA*gene in transgenic*Gladiolus*leaves. A**. Relative *uidA* mRNA levels. **B.** Specific GUS enzyme activity. Each bar represents a transformed plant line in A and B. Lines from left to right-G1-1: 1, 2, 3 and G1-3: 4, 5, 6. The black bar represents the average of the plant lines for each construct. GUS specific activity and *uidA* mRNA levels were measured using three plants for each line. The mean and standard error are shown. **C.** Specific GUS enzyme activity/unit *uidA* mRNA. Average GUS activity was divided by its corresponding mRNA level for each DNA construct.

**Figure 6:**
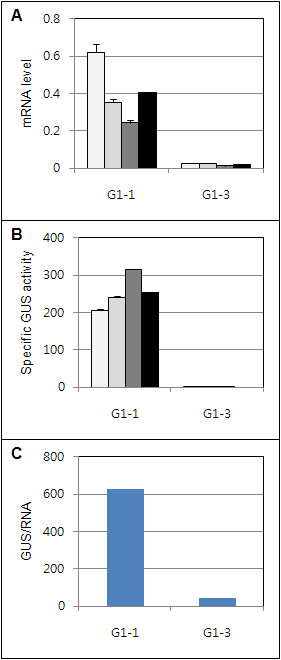
**Expression of the*uidA*gene in transgenic*Arabidopsis*leaves. A**. Relative *uidA* mRNA levels. **B**. Specific GUS enzyme activity. Each bar represents a transformed plant line in A and B. Lines from left to right-G1-1: 113, 117, 118, G1-3: 131, 132, 134, 35 S: 1, 2, 7. The black bars represent the average of the plant lines for each construct. GUS specific activity and *uidA* mRNA levels were measured using three plants for each line, and the mean and standard error are shown. **C**. Specific GUS enzyme activity/unit *uidA* mRNA. Average GUS activity of each construct was divided by its corresponding mRNA level for each DNA construct.

The two constructs, G1-1 and its intronless version G1-3, produce identical mRNAs that have the same nucleotide sequence in the 5′ leader. In leaves of transgenic *Gladiolus* plants the level of *uidA* mRNA was two-fold higher with the promoter only and its 5′UTR (G1-3) as compared to the 1.9 kb promoter that included the intron (G1-1) (Figure 5A). In comparison, *Arabidopsis* leaves transformed with G1-1 had significantly higher levels of *uidA* mRNA than leaves with G1-3 (Figure 6A). The intron affected the level of *uidA* mRNA in *Arabidopsis* but not in *Gladiolus*. Splicing of the intron appeared to be occur efficiently from the *GUBQ1* mRNA in leaves of *Gladiolus*. The trend in translation efficiency was similar for the two species (Figures 5C and 6C). In both *Gladiolus* and *Arabidopsis* the highest translation efficiency was with the 1.9 kb promoter (G1-1), and efficiency was negligible with the promoter and 5′UTR (G1-3).

**Figure 7:**
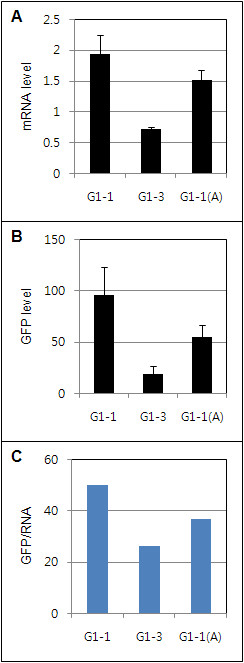
**Transient expression of the*gfp*gene in tobacco leaves. A**. Relative GFP mRNA level. **B**. Relative amount of GFP expressed. C. Specific GFP amount/unit mRNA of GFP. Average GFP level of three leaves for each construct divided by its corresponding mRNA level. G1-1(A) contained the *Arabidopsis actin7* gene and its 5′-UTR intron (540 bp) instead of the *Gladiolus* 5′-UTR intron.

**Figure 8:**
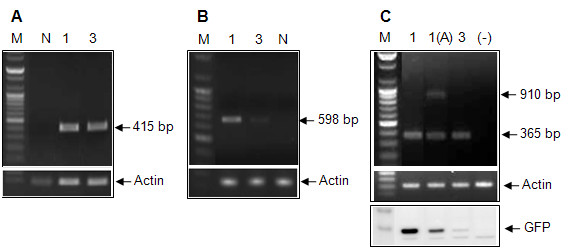
**RT-PCR analysis using the primers 5′UTR-exon F and*uidA*200 R/*gfp*R in (A) transgenic*Gladiolus*leaves (B) transgenic*Arabidopsis*leaves, and (C) transiently transformed tobacco leaves.** Western blot analysis of GFP shown in bottom panel of C. The vectors were pUC-GUS for *Gladiolus*, pCAMBIA for *Arabidopsis*, and pBI121 for tobacco. pCAMBIA contains “LacZ alpha, truncated” between the *GUBQ1*promoter and *uidA* gene”. Abbreviations: Non-transformed plants (N), G1-1 (1), G1-3 (3), G1-1 promoter with *act7* intron [1(A)], transient expression with pBI121 vector (−). *Actin* gene primers specific to each plant species analyzed were used as the reference gene to verify RT-PCR performance and the possibility of DNA contamination (bottom panel in A and B, middle panel in C).

The *act7* intron isolated from *Arabidopsis* was substituted with the *Gladiolus* intron in the G1-1 construct to determine the effect of a dicot intron rather than a non-cereal monocot intron on transient GFP expression and translation in tobacco. Although the *Arabidopsis act7* intron did not increase GFP expression to the same extent as the *Gladiolus* intron, expression with the *act7* intron was approximately half the level of G1-1 (Figure 7B), and there was a corresponding increase in GFP mRNA level (Figure 7A). The translation efficiency of the *GUBQ1* promoter was higher when either intron, the *Arabidopsis act7* or *Gladiolus GUBQ1* intron were present (Figure 7C). The level of GFP protein produced in tobacco was found to be significantly higher with the *Gladiolus GUBQ1* intron as compared to the *act7* intron (Figure 8C bottom panel).

It appears that in tobacco the *Arabidopsis act7* intron was not spliced as efficiently as the *Gladiolus GUBQ1* intron (Figure 8C top panel). Two bands were produced after RT-PCR with 1(A) primers for amplification of the *Arabidopsis act7* intron (Figure 8C top panel). The 365 bp band is expected when the *act7* intron is spliced out, and the second 910 bp band indicates that 1(A) splicing was incomplete. An *actin* gene specific to each species analyzed was used as a reference gene to verify RT-PCR performance and the possibility of DNA contamination (Figure 8A and B lower panel, C middle panel). RNA samples were not contaminated with DNA because only one band was present for *actin*. A second *actin* band would have resulted from DNA that did not have its *actin* intron spliced out (Figure 8A and B lower panel, C middle panel).

## Discussion

GUS expression in *Gladiolus* and *Arabidopsis* was dependent on the presence of the *GUBQ1* intron. Sequencing of this intron showed that its 68% AU content is more similar to that of dicots that contain a minimum of 60% AU and an average of 74% AU in comparison to cereal monocot introns that average 59% AU, and this may explain why the intron functioned well in *Arabidopsis* and tobacco. Splicing of introns is more precise in dicots than in cereal monocots, and the AT nucleotides are thought to facilitate splicing in dicots [19]. More recently Rose et al. [20] examined intron sequences in the *Arabidopsis thaliana* genome and concluded that sequences that enhance gene expression are dispersed throughout an intron, and these specific sequences are conserved between monocots and dicots. Deletion of the intron from three polyubiquitin promoters, *UBQ3UBQ10*, and *UBQ11* isolated from *Arabidopsis,* resulted in a 2.5–3× decrease in gene expression in *Arabidopsis* leaves [2]. Omission of the intron from the *GUBQ1* promoter resulted in a much more dramatic effect, no expression, in *Arabidopsis* leaves as compared to its omission from the *Arabidopsis*-derived polyubiquitin promoters.

The role of the intron in controlling gene expression is typically more critical in cereal monocots than dicots, and it appears to be critical in leaves of this non-cereal monocot, *Gladiolus*. Levels of GUS expression were reduced to background in rice when the promoter’s intron was absent from the rice *Act1* and *RUBQ2* promoters [21,22].

Studies with other non-cereal monocots have found that some, but not all, introns derived from cereal monocots may stimulate gene expression, and dicot-derived introns result in relatively lower levels of IME. A transient transformation study using lily and tulip leaves showed a decrease in GUS expression when the maize *Adh*1 intron was included with the CaMV 35 S promoter, and this same result occurred in sugarcane [11,23]. Chloramphenical acetyltransferase expression increased in protoplasts of *Panicum* and *Pennisetum* 23–28× following transient transformation with the CaMV 35 S promoter and first intron of the maize *shrunken-1* locus [13]. This same intron resulted in higher, 91×, CAT expression in maize protoplasts. The maize *Wx*, maize *Bz*, rice *Act1Adh1SShPetunia Chs*, and maize *Ubi1* introns were tested in bluegrass suspension cells using transient transformation, and the highest level of GUS expression, a 26X increase, was with the maize *Ubi* intron [12]. Transient expression using embryogenic cells of banana was enhanced 100×, 300X, and 10× when the maize *Ubi1*, rice *Act1*, and sugarcane *RbcS* introns were included with the banana bunchy top virus DNA-6 promoter, respectively [14]. The maize *Ubi1* intron with banana bunchy top virus DNA 6 promoter was used for stable transformation, and banana plants had levels of GUS expression similar to that of the CaMV 35 S promoter [14]. It is difficult to predict the level of IME for each non-cereal monocot species and a particular intron. The maize *Ubi1* and rice *Act1* promoters showed very low levels of transient GUS expression in *Gladiolus*[24] possibly because of their introns, yet these two introns showed high levels of IME, 100–300×, in banana and only 26 × in bluegrass.

Rose and Beliakoff [25] have shown IME using either of the first two *PAT1* introns. Because the levels of *uidA* mRNA increased during IME, but the rate of transcription remained constant, IME with the *PAT1* introns was concluded to occur through a post-transcription mechanism. In a later study using *Arabidopsis*, the position of the tryptophan biosynthesis 1 (*Trp1*) and *UBQ10* intron was varied in the coding and 3’UTR regions [26]. Both introns at all locations stimulated GUS expression more than mRNA accumulation. Results from this study support a model for introns operating at both the level of transcription and post-transcription. Rose’s recent study of IME [27] using hybrid introns in either their normal orientation or reverse complement resulted in a model of IME that acts at the level of the transcribed DNA rather than the nascent RNA. Samadder et al. [28] found that IME with the 5′UTR intron of the rice *Rubi3* gene increased the gene’s mRNA level 20X and stimulated the intron’s transcription 2X in a nuclear run-on experiment. Their results support both transcriptional and post-transcriptional effects of the rice *Rubi3* intron, and suggest that the main effect was at the level of post-transcription. In both *Gladiolus* and *Arabidopsis* the 5′UTR-intron stimulated translation efficiency supporting the effect of the 5′UTR-intron at the post-transcriptional level.

In mammalian cells, genes containing an intron generally show more expression than the same genes lacking an intron. Wiegand et al. [29] did experiments to separate the effects of intron removal and exon junction complex formation on gene expression and found that the components of the exon junction complex function to activate splicing in human cells. Removal of an intron will not increase gene expression without exon junction complex formation. An increased amount of protein translated in mammalian cells was associated with splicing of the protein’s mRNA [30]. In our experiments with GFP expression in tobacco, possibly the exon junction complex did not occur in all of the GFP RNAs resulting in incomplete splicing and less translation than with the *GUBQ1* 5′UTR-intron. Future research on the role of the exon junction complex formation in plant cells will be useful to elucidating the mechanism of enhanced gene expression associated with intron splicing.

## Conclusions

Levels of GUS expression were comparable in leaves of *Gladiolus* and *Arabidopsis* under the G1-1 and CaMV 35 S promoters. Removal of the 5′UTR-intron decreased expression in leaves of both species to background levels demonstrating the contribution of the 5′UTR-intron to expression. Translation efficiency was increased in both species with the presence of the 5′UTR-intron.

## Methods

### Plasmid construction

Isolation of the polyubiquitin promoter from *GladiolusGUBQ1*, (GenBank accession DQ445914) using the Genome Walker Kit (Clontech) was previously described [15]. The 1.9 kb *GUBQ1* promoter (G1-1) and a 0.68 kb intronless version of this promoter (G1-3) were previously subcloned into pUC-GUS and used for transient GUS expression in callus [15]. For *Arabidopsis* transformations, the *GUBQ1* promoter and its intronless version were subcloned into the binary vector pCAMBIA1391Z. For the transient expression assay of tobacco, either G1-1(A) or G1-3 were subcloned into the binary vector pBI121-GFP which contained the *gfp* gene instead of the *uidA* gene (Figure 1). G1-1 contains 1898 bp which consists of 600 bp promoter (Gubi1-P), 80 bp 5′UTR-exon (5′U-E), and 1218 bp 5′UTR-intron (5′U-I). In G1-1 (A), the 1218 bp 5′U-I of *GUBQ1* has been substituted with the *Arabidopsis actin7* gene 5′UTR-intron (541 bp).

The 5′UTR was identified using the 5′RACE & 3′RACE kit (Clontech). Total RNA was isolated from *Gladiolus* and 2 μg used for RT PCR according to the manufacturer’s directions. AP1, an adaptor-specific primer present in the kit, and a ubiquitin gene-specific primer (RT: G1-1 and AS: AGG AAT CCC CTC CTT GTC CTG G) were used for the first PCR reaction of 5′ RACE. The second PCR reaction used AP2, an adaptor-specific primer in the kit, and a ubiquitin gene-specific primer (RT:G1-2 and AS: ATC GAT TGT GTC GGA GCT CTC C). PCR products were cloned into a pTOPO-TA vector (Invitrogen) for sequencing. The 5′UTR sequence was revealed by sequencing and then verified by showing that this sequence was spliced out.

### *Gladiolus* transformation

Suspension cells of *Gladiolus* cv. Jenny Lee were transformed using the PDS-1000/He gene gun (Bio-Rad, Hercules, CA, http://www.bio-rad.com) [24]. Callus was initiated from plants growing in vitro on Murashige and Skoog’s medium (MS) [31] containing 3% sucrose, 1.0 mg/l glycine, 100 mg/l m-inositol, 1.0 mg/l thiamine, 0.5 mg/l pyridoxine, 0.5 mg/l nicotinic acid, 0.5 mg/l (2.2 μM) 2,4-dichlorophenoxyacetic acid (2,4-D), and 0.2% Phytagel (Sigma-Aldrich, http://www.sigmaaldrich.com). The callus was used to start suspension cells that were grown in the same medium lacking Phytagel. Both callus and suspension cells were grown in the dark at 25°C. Suspension cells were grown on a gyratory shaker at 120 rpm and transferred every 2 weeks by placing half of the cells into 30 ml of fresh medium.

Suspension cells were co-bombarded at 1200 psi (8.3 MPa) with p35SAc that contains the selectable marker gene phosphinothricin acetyltransferase (PAT) under control of the CaMV 35 S promoter (received from AgrEvo, Somerville, NY) and one of the promoter plasmid DNAs (CaMV 35 S, G1-1, or G1-3). One week after bombardment, cells were placed on MS medium as described for callus initiation supplemented with 3 mg/l bialaphos (Meiji Co., http://www.meiji.co.jp) for selection. Callus was transferred monthly to fresh selection medium, and after about 4 months, surviving callus pieces were transferred to regeneration medium. Regeneration medium is the same as callus initiation medium except with 2 mg/l (9.3 μM) kinetin rather than 2,4-D, and supplemented with 1 mg/l bialaphos. Plantlets recovered after growth on regeneration medium were grown on MS medium without hormones and without bialaphos. The cultures were grown under a 12-h photoperiod using cool-white fluorescent lights (40–60 μmol·m^2^·s^1^).

### *Arabidopsis* transformation

Transformation of *Arabidopsis thaliana* ecotype Columbia plants grown 5 weeks in a growth chamber with a 16 h photoperiod at 22°C were transformed with *Agrobacterium tumefaciens* strain C58C1 using vacuum infiltration [32]. Hygromycin-resistant T1 plants were selected by planting seeds on MS medium supplemented with 30 mg/l hygromycin. Hygromycin resistant seedlings were then transferred to soil and transgenic plants were verified to be transformed by PCR. The *gus* gene was amplified using the primer sequences 5′-GTT GGG CAG GCC AGC GTA TCG TG-3′ and 5′-TAA CCT TCA CCC GGT TGC CAG AGG-3′ and thermal cycler conditions of: 1 cycle of 95°C for 5 min and 35 cycles of 94°C for 30 s, 62°C for 30 s, 72°C for 1 min, followed by 3 min at 72°C.

### Agro-infiltration

*Agrobacterium tumefaciens* strain GV3101 was used for transient expression of tobacco (*Nicotiana benthamiana). Agrobacterium* was cultured for 16 h in liquid medium. Cells were then centrifuged and resuspended in 10 mM MgCl_2_ at an OD_600_ of 0.9. Resuspended *Agrobacterium* cells were vacuum infiltrated into tobacco leaves that had been collected from tobacco plants grown in soil at 22 ~ 24°C for 4-5 weeks under a 16 h light photoperiod. Tobacco leaves were harvested 3 days after vacuum infiltration and used for *gfp* expression analysis.

### RNA isolation and RT-PCR

RNA was isolated from plant tissues using the Qiagen RNeasy kit (Qiagen Inc, Valencia, CA). The isolated RNA was treated with RNase-free DNase (Roche) to remove DNA. Reverse transcription was performed using 1 μg total RNA and 20 μl reaction mix of the iNtRON Power cDNA synthesis Kit (Intronbiotech, http://www.intronbio.com) and AMV reverse transcriptase (Intronbiotech) according to the manufacturer’s directions. For identification of 5′UTR-intron splicing in transgenic plants, RT-PCR was performed with the 5′UTR exon specific primer, 5′U-E F (5′-AGG GTT TTC TCA TCC CCA AAT T-3′) and either a *uidA* or *gfp* gene specific primer, *uidA* 200 R (5′- CCT GAT GCT CCA TCA CTT CCT G-3′)/*gfp* R (5′-AGA AGA TGG TGC GCT CCT GG-3′). For the RT-PCR, 2 μl of each primer (0.2 mM final concentration), and 2 μl of the cDNA preparation were used in the reaction mix (25 μl final volume). The thermal cycler conditions of: 1 cycle of 95°C for 5 min and 30 cycles of 94°C for 20 s, 55°C for 20 sec, 72°C for 1 min. RT-PCR products were cloned into a pTOPO-TA vector (Invitrogen) for sequencing. Quantitative PCR amplification was performed with gene-specific primers and the SYBR Green Master Mix kit (Qiagen) using a Rotor-Gene 6000 real time amplification operator (Corbett Research, Mortlake, Australia). The reaction mix (25 μl final volume) consisted of 12.5 μl of the SYBR Green PCR Master Mix, 2 μl of each primer (0.2 mM final concentration), and 2 μl of the cDNA preparation. The thermal cycler conditions of: 1 cycle of 95°C for 15 min and 45 cycles of 94°C for 20 s, 55°C for 20 sec, 72°C for 30s.

In real-time PCR, the *actin* gene specific to each plants species being analyzed was used as the reference gene and to determine the possibility of DNA contamination. The *actin* primer sequences included the following: 1) for the *Gladiolus actin* gene 5′-CTG CCA TGT ATG TTG CAA TCC A-3′ and 5′-GGA AGA GGA CTT CAG GGC ACC TG-3′ (384 bp), 2) for the *Arabidopsis actin* gene 5′-GTC CCT GCC ATG TAT GTT GCC-3′, and 5′-GTG GTG AAC ATG TAA CCT CTC-3′ (200 bp), and 3) for the *Nicotiana actin* gene 5′-TTG GAA TGG AAG CTG CTG GA-3′, and 5′-TCA GGA GGC GCC ACC ACC TT-3′ (202 bp).

All Samples were run three times in triplicate.

### *Arabidopsis Actin7 gene 5’-UTR intron isolation*

The 5′-UTR intron of *actin7* gene (Genebank ID, U27811) was isolated from genomic DNA of *Arabidopsis thaliana* ecotype Columbia. PCR amplification of the 5′-UTR intron was accomplished using the primer sequences 5′-AGG TGA GTC TCT AGA TCC G-3′ and 5′-CCT AAA AAA AAA GTA AAA TGA AAC-3′ and thermal cycler conditions of: 1 cycle of 95°C for 5 min and 35 cycles of 94°C for 30 s, 50°C for 30 s, 72°C for 1 min, followed by 3 min at 72°C. PCR products were cloned and sequenced.

### GUS expression

The specific activity of GUS expression was determined by fluorometric determination of methylumbelliferone (4-MU) [33]. The extraction buffer consisted of 100 mM KH_2_PO_4_, pH 7.0 and 1 mM dithiothreitol. Extracts were centrifuged at 16,000 × *g* at 4°C for 12 min, and a 50 μl aliquot was added to 0.5 ml of assay buffer (1 mM methylumbelliferyl-β-D-glucuronide) for incubation at 37°C. A 100 μl aliquot of the assay buffer containing cell extract was added to 0.9 ml of 0.2 M sodium carbonate after incubation for different lengths of time. Fluorescence of 4-MU was measured using a Bio-Rad VersaFluor Fluorimeter set at 360/40 nm excitation and 460/10 nm emission. The amount of protein in the cell extract was determined using the bicinchoninic protein assay reagent (Pierce, http://www.piercenet.com).

Transgenic plants were assayed for expression of the *gus* gene that codes for β-glucuronidase (GUS) following the histochemical staining procedure described by Jefferson et al. [33] with some modifications. Shoots were incubated 16 h at 37°C in staining solution [100 mM sodium phosphate, pH 7, 0.5 mM potassium ferricyanide, 0.5 mM potassium ferrocyanide, 10 mM Na_2_EDTA, 0.5% (v/v) Triton X-100] with 0.5 mg/l 5-bromo-4-chloro-3-indoly-β-D-glucuronide. After 16 h of staining, the shoots were cleared and fixed in a solution of 75% (v/v) ethanol:1% (v/v) acetic acid.

### Statistics

Three independently transformed plant lines of *Gladiolus* growing in vitro or three lines of *Arabidopsis* were analyzed for each DNA construct, and three plants were analyzed for each transgenic line. Three plants of each plant line were used for the mean determination of GUS expression and standard error calculated.

For real time PCR the relative amount of target RNA for each sample was calculated by the statistical analysis method [34].

### Southern hybridization

Genomic DNA for Southern hybridizations was isolated from plants using the DNeasy Plant Mini Kit (Qiagen) according to the manufacturer’s instructions. Genomic DNA was digested with *Hind III*, electrophoresed on a 0.8% agarose gel in 0.5× TBE buffer, and then transferred to a nitrocellulose membrane. For hybridization a 492 bp PCR-amplified *gusA* gene product was labeled using the PCR DIG Probe Synthesis kit (Roche, http://www.roche.com) and used as the probe. Hybridization and detection of the DIG labeled nucleic acid was performed using the DIG Easy Hyb and DIG Nucleic Acid Detection kits (Roche).

### Western blot

Plant leaves were ground in Bradley buffer (50 mM Tris–HCl pH 7.5, 10 mM KCl, 20% glycerol, 0.4 M sucrose, 5 mM MgCl_2_, 10 mM β–mercaptoethanol, 1 mM PMSF) and centrifuged for 20 min at 13,000 × g, 4°C. The supernatant was used for SDS-PAGE gel electrophoresis, and then transferred onto a polyvinylidene difluoride (PVDF) membrane. The membrane was incubated with blocking solution (Tris-buffered saline solution pH 7.4, 0.1% Tween 20, 5% nonfat milk). The GFP detection rabbit anti-GFP polyclonal antibody was used as the primary antibody, and anti-rabbit horseradish peroxidase-conjugated was used as the secondary antibody. Immunobiochemical detection was performed using the ECL Plus detection system (Millipore). For quantification of GFP the Multi Gauge Version 3.0 program was used following exposure of the Western blot to Fuji film.

## Authors’ contributions

KK conceived the idea of studying the effect of the *GUBQ1* intron on gene expression in *Gladiolus* plants, did the *Gladiolus* expression studies, and wrote the manuscript. YHJ conceived the idea of studying the effect of the *GUBQ1* intron on gene expression in *Arabidopsis.* AY Kim did the real-time PCR and *tobacco* transient assay analysis. SHP did the GFP analysis. All authors read and approved the final manuscript.
